# Is There a Link between Basal Metabolic Rate, Spleen Volume and Hepatic Growth Factor Levels in Patients with Obesity-Related NAFLD?

**DOI:** 10.3390/jcm8101510

**Published:** 2019-09-20

**Authors:** Giovanni Tarantino, Vincenzo Citro, Paolo Conforti, Clara Balsano, Domenico Capone

**Affiliations:** 1 Department of Clinical Medicine and Surgery, Federico II University Medical School of Naples, 80131 Napoli NA, Italy; 2 Department of General Medicine, “Umberto I” Hospital, Nocera Inferiore (Sa), 84014 Nocera Inferiore SA, Italy; v.citro@libero.it; 3 “Federico II” University Medical School of Naples, 80131 Napoli NA, Italy; pconforti8@gmail.com; 4 Department of Clinical Medicine, Life, Health & Environmental Sciences-MESVA, University of L’Aquila, 67100 L’Aquila AQ, Italy; clara.balsano@cc.univaq.it; 5 Care Department of Public Health and Drug-Use, Section of Medical Pharmacology and Toxicology, “Federico II” University, 80131 Naples NA, Italy; docapone@unina.it

**Keywords:** NAFLD, obesity, HGF, calorimetry indices, spleen

## Abstract

Background: Recent pieces of research point to a link between basal metabolic rate (BMR) and non-alcoholic fatty liver disease (NAFLD) or hepatic steatosis (HS). The spleen in obese patients is associated with the cardiovascular system. Enlargement of the spleen is suggestive of nonalcoholic steatohepatitis (NASH). Patients with NASH present an increase in growth factor (HGF) as well as those with advanced heart failure. Interleukin-16 and interleukin-12p40 levels were found to correlate significantly with BMI, and waist circumference. Aim: We tried to find a relationship between BMR, spleen length and HGF. Methods: We analysed retrospective data from 80 obese patients with NAFLD. We evaluated indices of indirect calorimetry by the bioimpendance analysis; carotid intima-media thickness (IMT), spleen length (SLD) and HS by ultrasonography; serum HGF, IL-16, IL-12p40 and IL-6 concentrations by a magnetic bead-based multiplex immunoassays and the severity of NAFLD by BARD score > 2. Results: HGF levels of the obese were higher than those of controls, *p* < 0.001. At linear regression, BMR was foreseen by spleen length (*p* < 0.001), which was predicted by HGF (*p* = 0.04). BMR was predicted by IL-16 (*p* = 0.005), which predicted HGF, *p* = 0.034. Only fat mass, among other factors, predicted early atherosclerosis, *p* = 0.017; IL-12p40 did not predict IMT, HGF and BMR (*p* = 0.57, 0.09 and 0.59, respectively). The BARD score > 2 was negatively predicted by BMR and FFM (*p* =0.032 and 0.031, respectively), at the logistic regression. Interesting findings at the extended regression (mediation effect) were: IL-16 was likely causal in predicting BMR by HGF levels; HGF was influential in predicting BMR by SLD level. HS was predicted by SLD in males (*p* = 0.014), of advanced age (*p* < 0.001) and by BMR (*p* < 0.001). IL-6 concentrations, but not BMR were influential in the prediction of HS by SLD. Conclusion: These data reinforce the concept that the immune system is a sensor of the metabolic state, showing a link between HGF levels and BMR, which is mediated by IL-16 (cytokine inducing a cascade of inflammatory factors), and ascertaining the influential effect of the spleen, as main immune organ.

## 1. Introduction

There is a wide consensus about the immune system as a sensor of the metabolic state [1]. In a pioneering study, obese subjects showed lower resting metabolic rate (RMR) than control subjects [2], but recently this finding is challenged [3]. In recent year, RMR has been substituted by the more precise basal metabolic rate (BMR), which depends on body composition as expressed by fat-free mass (FFM) and fat mass (FM) [4,5]. In this context, increased BMR may be a clue of MS when dealing with hepatic steatosis (HS) or nonalcoholic fatty liver disease (NAFLD) patients [6]. Furthermore, a regression of NAFLD following improvement of the increased BMR was clearly showed [7].

Novel results highlight the important contribution that spleen has on resting energy expenditure (REE), synonym of RMR [8]. Spleen, was found enlarged at significant levels (38%) in obese patients [9]. Indeed, splenic enlargement is well detected by ultrasonography as length of the organ [10]. The association between spleen and cardio vascular (CV) system is tested by multiple studies, providing evidence of a cardio-splenic axis in humans [11,12,13,14]. 

Similarly, a liver-spleen axis has been recently proposed in the sense that increased spleen volume—a stable index of chronic inflammation and activation of the immune system—is suggestive of advanced HS [15,16]. Hepatic growth factor (HGF), secreted by mesenchymal cells, has one of its expressions in the spleen [17], acting via both a paracrine and an endocrine mechanism [18], specifically through its interaction with c-met receptor as regulator of immune cell functions [19]. HGF plays a role in regulating the chemotaxis of T cells into heart tissue [20,21,22]. Still, plasma from patients with advanced heart failure presents elevated levels of HGF [23]. On the other hand, patients with the most severe form of NAFLD, i.e., nonalcoholic steatohepatitis (NASH) present a significant increase in circulating levels of HGF [24].

Authors found that the increased levels of f interleukin-(IL)-16 and those of IL-12p40 correlated significantly with BMI, and waist circumference (WC) [25]. Northern blotting has identified IL-16 mRNA predominantly in spleen. [26]. In this milieu IL-12p40 could induce the expression of IL-16 [27,28]. At the best of our knowledge, no research raised a question as to whether BMR can actually be linked to some immune system parameters such as spleen as well as to L-16, IL-12p40 and HGF in obesity-related NAFLD patients. Similarly, there are no literature data on the relationship between atherosclerosis, evaluated as carotid intima-media thickness (IMT) and the afore mentioned factors of immunity and/or indirect calorimetry indices. As key aim, we tried to verify whether the correlation between the NAFLD severity and spleen was mediated by IL-6, a mediator of a number of chronic inflammatory conditions.

Pursuing the objective of clarifying these aspects, at the light of the previously cited studies, which permitted somehow to hypothesise this intriguing association, we analysed in a retrospective way data from obese patients with low prevalence of co-morbidities, but NAFLD.

## 2. Method

### 2.1. Patients

We carried out a cross-sectional type of observational (retrospective) study where at a particular point of time we analysed characteristics of obese patients without follow-up, using as main variables: HGF, IL-16 and IL-12p40 levels, IL-6 concentrations, spleen length, IMT values, indirect calorimetry indices and HS scores. Specifically, this sub-study used the same original patient sample contained in a previous research [29], but with completely different analytical/statistical approaches resulting to be equally valid, [29].

### 2.2. Inclusion Criteria 

Eighty obese patients of different grade of obesity, on calorie-reduced, low-fat diet and sedentary lifestyle, with low prevalence of co-morbidities, such as type 2 diabetes mellitus and hypertension but NALFD, US-documented, were retrospectively re-evaluated.

### 2.3. Exclusion Criteria

We excluded patients from this recent evaluation if, at the time of blood specimen collection, they had self-reported present or antecedent unexplained muscle trauma in the past months or minor/major surgery or other chronic disease, such cancer or cardiovascular events or H. pylori infection (IL-16) or lung fibrosis (HGF) and the use of medications that might have affected body composition or muscle metabolism (e.g., steroids) or could have affected inflammation response.

Furthermore, viral, autoimmune, metabolic liver diseases (Wilson disease, hemochromatosis or antitrypsin deficiency) were ruled out by using appropriate testing, according to well-accepted diagnostic guidelines. Celiac disease was excluded by evaluating IgA anti-tissue transglutaminase antibodies. Alcohol abuse was disallowed, following the DSM-IV diagnostic criteria, by means of screening tests such as MAST (Michigan Alcohol Screening Test) and CAGE (Cut down, Annoyed, Guilty, and Eye opener), as well as random tests for blood alcohol concentration and the use of a surrogate marker, e.g., Mean Corpuscular Volume. Patients on anti-hypertensive drugs, and those treated with metformin or insulin, maintained a balanced therapeutic regimen throughout the study.

### 2.4. Anthropometric Evaluation

We established the three classes of obesity (light, moderate, and severe or I–II–III) on the basis of BMI cut-off points of 30–34.9 and 35–39.9 and > 40 kg/m^2^, respectively.

Visceral obesity was identified by measuring WC at the midpoint between the lower border of the rib cage and the iliac crest. Hip circumference was measured around the widest part of the buttocks, with the tape parallel to the floor, and the waist-to-hip ratio (WHR) was calculated. 

### 2.5. Metabolic Assessment

The Adults Treatment Panel III was selected to ascertain the presence of the metabolic syndrome (MS), considering at least three criteria: plasma glucose concentrations ≥ 100 mg dL^−1^, WC >102/88 cm (male/female), serum HDL concentration < 50 mg dL^−1^ for women and < 40 mg dL^−1^ for men, blood pressure ≥ 130/85 mmHg, and serum triglyceride concentration ≥ 150 mg dL^−1^. Furthermore, we applied the criteria for Europids following the International Diabetes Classification (IDF). Accordingly, patients were defined as having the metabolic syndrome if they showed central obesity (measured as WC with ethnicity specific values, e.g., for males and females equal or superior to 94 and 80 cm, respectively), plus any two of the following four factors: Triglycerides > 150 mg/dL or specific treatment for this lipid abnormality; cholesterol HDL < 50 mg/dL for females and 40 mg/dL for males or specific treatment for this dyslipidemia; systolic and diastolic blood pressure equal or superior to 130 and 85 mmHg, respectively; fasting plasma glucose > 100 mg/mL or previously diagnosed type 2 diabetes mellitus. International Diabetes Federation, 2007; IR was evaluated by the HOmeostatic Metabolic Assessment (HOMA) method employing the formula: fasting insulin (μU/mL) * fasting glucose (mg/dL)/405 [30].

### 2.6. Laboratory Data

HGF, IL-6, Il-16 and IL12p40 levels of 78 patients derived from a previously studied 48-cytokine/chemokine panel [31], which was performed on serum samples using a magnetic bead-based multiplex immunoassays (Bio-Plex) (BIO-RAD Laboratories, Milano, Italy) following manufactures’ instructions. Data from the reactions were acquired using the Bio-Plex 200 reader, while a digital processor managed data output and the Bio-Plex Manager software returned data as Median Fluorescence Intensity (MFI) and concentration (pg/mL).

HGF levels of the obese were compared to those of 33 controls.

To assess liver fibrosis, we used the Bard score. In brief, three variables were combined in a weighted sum (BMI > or = 28 = 1 point, AST/ALT ratio of > or = 0.8 = 2 points, T2DM = 1 point) to form an easily calculated composite score for predicting advanced fibrosis. A score of 2–4 was associated with an OR for advanced fibrosis of 17 (confidence interval 9.2 to 31.9) and a negative predictive value of 96% [32]. The hs-CRP values, determined by the ELISA test, with reference values between 0.03 and 0.86 mg/dL in healthy men and between 0.02 and 0.91 mg/dL in healthy women (BioCheck, Inc., San Francisco, CA, USA) were assayed in collected and aliquoted samples and frozen at −20 °C. 

### 2.7. Ultrasound Features

Ultrasound (US) measurements were obtained by an Esaote (Genoa, Italy) system.

We chose the spleen length, assessed as spleen longitudinal diameter (SLD)—measured on longitudinal scan and carried out by the postero-lateral approach—to evaluate the spleen volume. The classification of “bright liver” or hepatic steatosis (HS) was based on the following scale of hyperechogenity: grade 0 = absent, 1 = light, 2 = moderate, 3 = severe, pointing out the difference between the densities of the liver and the right kidney [33], using a Convex Probe, with access to the liver through intercostal spaces along the mid-axillary line.

Visceral Adipose Tissue (VAT), was evaluated at Ultrasound and defined as the distance between the anterior wall of the aorta and the internal face of the recto-abdominal muscle perpendicular to the aorta, measured one cm above the umbilicus (when the aortic walls were obscured by bowel gas, a Doppler scan was used to detect VAT) [34].

IMT was defined as the distance between the ultrasound interfaces of the lumen-intima and media-adventitia. Six manual measurements were performed, with automatic border detection, at equal distances along one cm on the far wall of the common carotid, according to the consensus statement from the American Society of Echocardiography Carotid Intima-Media Thickness Task Force, endorsed by the Society for Vascular Medicine [35].

### 2.8. Indirect Calorimetry

RMR was measured by indirect calorimetry using a canopy system (V max 29 N, Sensor Medics, Anaheim, CA, USA) in a quiet environment and with patients in the supine position for 30 min before computation. After a 15–20 min adaptation to the instrument, oxygen consumption and carbon dioxide production were determined for 45 min. Energy expenditure was derived from CO_2_ production and _O2_ consumption by the means of the Weir formula neglecting protein oxidation [36]. BMR, expressed as kcal/24 h, was adjusted for changes in FFM, which was evaluated by single-frequency bioimpedance analysis (BIA) obtaining a RMR/FFM ratio, expressed as kcal/24 h*kg of body. FM and FFM percentage were estimated using the device’s standard built in prediction equations and were displayed on the machine and printed out [6].

BIA evaluation was performed between 10:00 a.m. and 4:00 p.m. The participants were required to fasting and avoiding vigorous exercise for at least 1h before BIA assessment. BIA measurements were performed while patients stood barefoot on the metal surface of the device and kept their arms loose and in parallel with the body. Measurement took 1–2 min(s) for each patient, and results were automatically printed out from the device. FM, FFM, and total body water were measured by BIA (6). The measurements were recorded by well-trained staff, using a BIACorpus RX 4000 (Medi-Cal Healthcare GmbH, Karlsruhe, Germany).

### 2.9. Statistics

Data, derived from a normally distributed population, were given as mean plus SD. Variables not normally distributed or ordinals are expressed as median plus 25–75 interquartile range (IQR). The difference in medians was assessed by the Mann-Whitney test. 

Frequency tables and multinomial logistic regression was used to assess relationships between obesity classes and MS presence.

To study predictions, we carried out various types of regression techniques. The linear regression analysis (ordinary least squares or OLS) as univariate analysis was employed evaluating the coefficient with its standard error, 95% confidence intervals (CI), the t (t-value) and squared-R. An ordered probit model was arranged to estimate predictions between an ordinal dependent variable, i.e., HS at US, expressed as severity grades (1–3) and a set of independent variables. The output showed the coefficients, their standard errors, the z-statistic (also called a Wald z-statistic), and the associated *p*-values. In suspicion of heteroscedasticity and having detected the presence of few outliers, we analyzed the correlation by the robust regression, using Least Absolute Deviations (LAD) Regression. 

Remaining unclear whether prediction represents unidirectional or bidirectional causality, to try to partially overcome this aspect, endogenous variables were studied.

A variable is said to be endogenous within the causal model if its value is determined or influenced by one or more of the independent variables, [37]. A purely endogenous variable is a factor that is entirely determined by the states of other variables in the system. An extended regression that fits a linear regression model that accommodates any combination of endogenous covariates was run ad hoc to test a possible mediation effect. Still, we evaluated at multiple linear regression the so called β or Beta factor, in order to study which of the independent variables have a greater effect on the dependent variables. 

Dealing with a binary dependent variable the prediction tool carried out was the logistic regression by which the Odds ratio with related 95% CI was evaluated, using as categorical variable BARD > or < 2.

In order to evaluate the degree of pair observations at US, we adopted the concordance correlation coefficient (ρ_c_), which measures precision and accuracy.

The power of this study was calculated on the difference of means of HGF levels between the obese and control group. To further deepen this aspect, a power analysis was performed using a slope test in the linear regression between the HS (d.v.) levels and the SLD (i.v.) values in the whole population of obese patients. 

Stata15.1 was the program on which we run statistics.

## 3. Results

### 3.1. Prevalence

**Figure 1:**
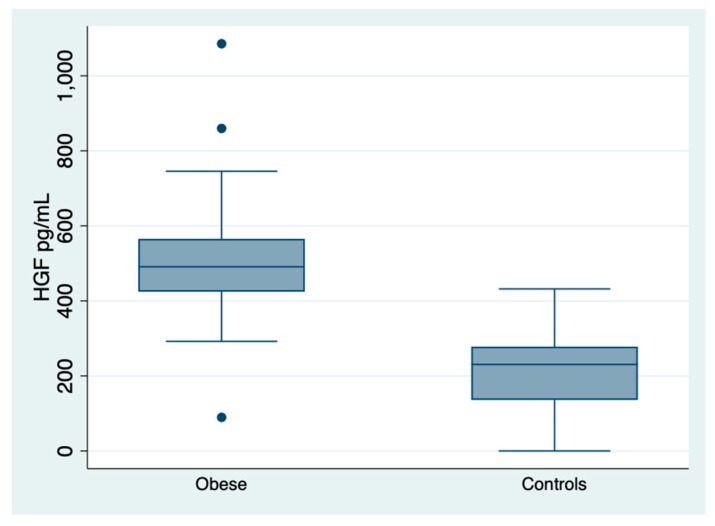
Growth factor concentrations in the obese and controls. Legend to Figure 1: HGF, hepatic growth factor. It is noteworthy to stress that the outliners were very few and that there is scarce overlapping between groups.

First of all, the median HGF levels of the obese population resulted to be higher than that of 33 controls, i.e., 491 (425–565) versus 230 (136–278) pg/mL; two-sample Wilcoxon rank-sum (Mann-Whitney) test *p* < 0.001, Figure 1. Concentrations of HGF in our controls were quite similar to the reported Biorad normal values, i.e., median 195, [38].

The obese population was characterised by normal or slightly elevated liver enzymes, mainly confronting the values of ALT (key enzyme in NAFLD patients) to the healthy upper limits, evidenced in [39]. Other clinical, laboratory and instrumental characteristics are shown in Table 1.

There was no difference in frequency when relating obesity classes to MS presence, Table 2.

Concerning the main inflammatory responses, the median CRP concentration was 0.56 (0.27–1.30) mg/L, specifically in females 0.55 (0.34–1.38) and males 0.59 (0.23–1.22), being only the third quartile superior the normal range values. 

### 3.2. Predictions

The first step was to find any association between calorimetric indices and some parameters of immune system. The results evidenced a link between the spleen volume, assessed as its length, and both BMR and FFM. Furthermore, IL-16 concentrations predicted BMR, Table 3.

A further step consisted in discovering any correlation between the early atherosclerosis, evaluated as IMT, and immune system parameters and indirect calorimetry indices taken into account in this study. As expected the role of adiposity was clearly evidenced in the sense that IMT was predicted by FF, Table 4.

Homing in on the concentrations of HGF, this growth factor was strongly associated with two main immune system parameters such as the spleen length and concentrations of IL-16, Table 5.

Having found a link of IL-16 with both BMR and HGF, we asked ourselves whether IL- 16 could play an influencing role in the association between BMR and HGF, by adopting the extended regression, powerful technique to discover an endogenous variable. The statistical output confirmed the likely causal role of IL-16 concentrations, Table 6.

Furthermore, having found a link of HGF with both BMR and SLD, we asked ourselves whether HGF concentrations, could play an influencing role in the association between BMR and the spleen length, evaluated as SLD, by using the same extended regression. The statistical output confirmed the likely causal role of HGF concentrations, Table 7.

Suffering the obese patients from HS, as per our selection, we tried to find some correlations between the severity of the liver fat, expressed as grades of HS, and spleen length, BMR and the two previously studied cytokines, i.e., IL-16 and IL-12p40. Only, SLD and BMR predicted HS at US, Table 8.

**Figure 2:**
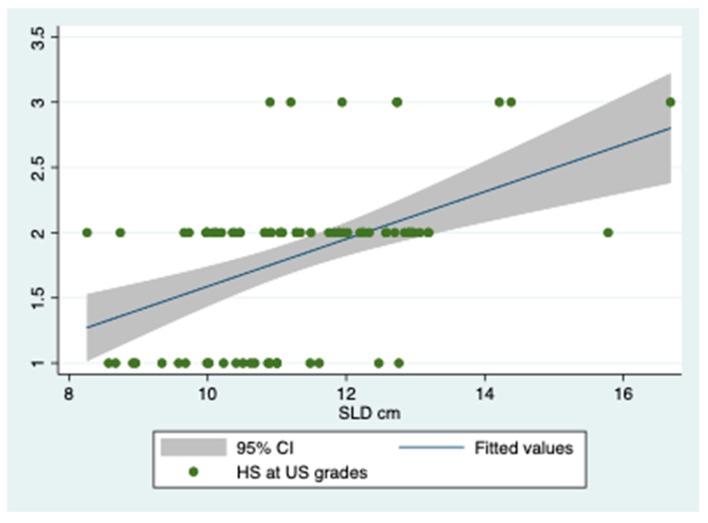
Association between spleen length and severity of hepatic steatosis. Legend to Figure 2: SLD, spleen longitudinal diameter; HS at US, hepatic steatosis at ultrasonography.

At multiple linear regression, among SLD, BMR, IL-16 and IL-12p40, only SDL remained as predictor of HS severity with Coef., Std. Err., z, *p* > |*z*|, (95% Conf. Interval) and **Beta** of 0.35; 12; **2.89**; 0.004;.11–58 and **36**, respectively. The graph of this regression is shown in Figure 2.

Coming back to the order probit regression, robust, evaluating the prediction of HS by SLD, inserting the interaction term, e.g., **i.Gender#c.HS**, only HS of males was predicted by SLD (*t* = 2.55, *p* = 0.014). Furthermore, inserting the interaction term, e.g., **c.HS#c.Age**, the age of patients played a strong role in predicting HS by SLD (*t* = 4.83; *p* = 0.000). Speculating on the role of BMR in predicting HS by SLD, we did not find that BMR was a mediator, Table 9.

The lack of correlation estimates var (e.HS) tells us about the absence of endogeneity in our model of extended linear regression, i.e., BMR is not an endogenous variable, not playing likely a causal role. The Prob > chi2 was significant and the F-statistic (Wald chi square) against the null, i.e., the excluded endogenous variable are irrelevant in the first-stage regression, was larger than 10, demonstrating that instruments/covariate/endogenous/explanatory variables are weak according to Staiger and Stock’s Rule of thumb; in our case is < 10; d.v., dependent variable; e.v., endogenous variable; i.v., independent variable. It noticeable the absence of significance in the correlation estimates, although both the regressions were significant, indicating that the independent variable, i.e., SLD predicts the dependent variables, i.e., HS and BMR, independently one from the other.

Having found that BMR does not influence the link between spleen volume and the grade of severity of HS, detected at ultrasonography, a successive step was to assess a possible association between spleen and HS studying other cytokines, which could play a significant role in modulating the interplay between this main immune organ and liver. The choice fell on IL-6, which demonstrated to be high influential, Table 10.

Finally, to ascertain which among HGF, SLD, BMR and FFM predicted BARD score > 2, a logistic regression was set, only BMR and FFM foreseeing the presence of advanced liver disease, evaluated by the afore mentioned score, Table 11.

As collateral finding, a strong association was found firstly between HS and IMT, and secondly between HOMA values and HS at US, by linear regression (robust) with Coef., Std. Err., *t*, *p* > |*t*|, (95% C.I.) = 0.017, 0.005, **3.15**, **0.002**, 0062, 0.027, respectively and by ordered profit regression (robust) with Coef. Std. Err., z, *p* > |*z*|, (95% C.I): 0.13, 0.032, **4.02**, **0.000**, 0.066, 0.19, respectively.

Furthermore, a significant correlation was found between the VAT and IMT, as evident in the following results of linear regression (robust), Coef., Std. Err., *t*, *p* > |*t*|, (95% C.I.) = 0.0045, 0.0013, **3.33**, **0.001**, 0.0018, 0.0071, respectlively.

The intra/inter-observational variability of US estimations was not significant, the mean difference being 1.9%, 2.2% and 3.1%, and 3.9%, 3.3% and 3.4% for the HS, VAT and SLD, respectively, with a ρc of 0.91. 

The power of this research was carried out by measuring the difference of means and SDs of HGF-β in the obese and controls groups, i.e., 501.46 ± 134.75 and 222.63 ± 78.05. The study turned out to be sufficiently powered (alpha= 0.001, power= 0.95) considering the sample size of the two studied populations (estimates sample size: total n 28, n 14 for group). Furthermore, the power analysis for a slope test in a simple linear regression, with a significant level of 0.001 and a power of 0.90 gave as estimated sample size n 74, using as the null slope the value of 0.4276 (Coefficient found in the regression: SLD—> HS) and as the alternative one = 0.

## 4. Discussion

In a retrospective way we studied the relationships between the energy expenditure of patients with obesity, likely characterised by a systemic, low-grade inflammatory process and some parameters of immune response, mediated by spleen, to try to explain the gap between HGF, increased in NAFLD patients and an early stage of cardiovascular disease, i.e., atherosclerosis. In this context, increased BMR may be a clue of MS [6] and NAFLD is a further expression of MS. 

The core findings of this research could be summarised in: (j) HGF levels of the obese were higher than those of controls; (jj) BMR was foreseen by spleen length, which in turn was predicted by HGF; (jjj) BMR was predicted by IL-16, which predicted HGF; (jjjj) FM predicted early atherosclerosis; (jjjjj) IL-12p40 did not predict IMT, HGF and BMR. 

Interestingly, the BARD score > 2 was negatively predicted by BMR and FFM. IL-16 was likely causal in predicting BMR by HGF levels. HGF was influential in predicting BMR by SLD level. 

Still, HS was predicted by SLD in males of advanced age and by BMR. IL-6 concentrations, but not BMR were influential in the prediction of HS by SLD. 

Concerning the BARD score, only 36 patients (45%) were classified as likely having the most severe form of NAFLD. Differently from NAFLD fibrosis score, this simple score bears the advantage of not ending up in a percentage of patients falling in the indeterminate category. 

It is noticeable that the severity of HS at US in the obese was inversely associated with FFM hypothesising that exercise and rate of weight loss influence these patients.

What about the relationships to early atherosclerosis, evaluated as IMT? IMT, among the afore mentioned cytokines, IL-16 and IL-12p40, the spleen length and indirect calorimetry indices, was associated only with FM, according to numerous studies linking adiposity, mainly abdominal, with a high cardiovascular risk. 

It is found that excessive adipose tissue mass (FM) as well as a (partial) lack of adipose tissue predisposes towards chronic cardio-metabolic diseases. This implies that total adipose tissue mass is not the predominant factor that explains the increased metabolic risk in obese individuals. Rather, the location where the excessive calories are stored in combination with adipose tissue function seem to determine metabolic health [40]. Also HGF is not related to IMT, reinforcing the finding that this growth factor as well as spleen play a role in the advanced stage of cardiovascular disease and not in the early one.

Unexpectedly, we found a correlation between HGF and IL-2 receptor-alpha as well as between HGF and MIP-1b (data not shown), but their significance should be further deepened.

How do our results fit with existing literature? In a study of 28 patients researchers found that serum HGF levels were higher in NASH patients than in the controls, although it was statistically insignificant and a correlation with MS could not be detected [24]. Our data are in discordance with the previously mentioned research, because there was no significant difference of HGF levels between the more and less severe form of HS (performed on BARD score > 2). Nevertheless, we stress that HGF levels predicted both severity of HS at US and spleen length that is a clue for advanced NAFLD.

Indeed, other pieces of evidence are steadily accumulating, in the sense that HGF induces an antioxidant response in hepatic cells, countering oxidative stress, main component of NAFLD [41]. Furthermore, deletion of HGF (hepatocyte growth factor)/mesenchymal-epithelial transition factor (c-Met) receptor leads to the development of severe NASH in mice [42]. Finally, HGF has a key role in insulin resistance, mechanism central to NAFLD onset and progression [43].

On the contrary, in mice HGF improves IR and prevents high-fat-diet-induced obesity [44]. Trying to explain the causal role of IL-16 in predicting BMR by HGF levels, we draw the attention on pathological conditions having overlapping mechanisms with NAFLD. Authors found that IL-16 levels significantly correlate to the disease activity of psoriasis, the duration of which is associated to spleen volume [45,46]. IL-16 might play an important role in the inflammatory process of patients suffering from acute myocardial infarction and correlates with inflammatory cell activation [47]. IL-16 is constitutively present in peripheral blood monocytes and spontaneously released during apoptosis [48].

About IL-12p40, we found no significant associations with early atherosclerosis and/or the studied parameter of immune system, i.e., HGF and/or the BMR. Vice versa, having evaluated the influence of spleen, assessed as SLD, in predicting BMR by HGF levels, we confirm the role of this organ in relation to energy expenditure and immune system.

As final consideration, the energy expenditure, observed in obese patients as a consequence of a systemic, low-grade, inflammatory process, may explain progression from obesity to MS, independent of the presence of NAFLD. I should point out that excess adiposity per se, even without accompanying metabolic health status, may contribute to fibrosis progress [49]. Recent data confirm that both FFM and FM are significant contributors to BMR [50]. 

## 5. Limitations

The findings in the current analysis nevertheless had a variety of caveats of their own, including the fact that, due to the characteristics of prediction (its bi-directionality), we do not know for sure which is the cause or the effect but this intertwined link provides a thought-provoking example of how apparently different biological properties could interact to determine a unique abnormal condition of health. Liver biopsy to assess the severity of NAFLD was nor originally performed for ethical reasons (absence of frank cytolysis in the obese) and technical difficulty due to the abundant adipose tissue. Notably, because of the retrospective nature of the study, we analysed only the available data. Finally, these data should be confirmed by prospective studies.

## 6. Conclusions 

We have found a link between BMR, spleen volume and HGF levels in patients with obesity-related NAFLD. Noticeably, spleen as an immune organ, even though it plays a role in advanced cardiovascular disease, was of scarce impact in the subclinical stage of atherosclerosis. 

Regardless, there is a long road ahead in demonstrating how HGF works in relation to the spleen, and there is much to understand what and how this growth factor is linked to BMR. That said, the strides in immunology in recent years make it tempting to speculate that growth factors may play an increasing role in modifying the progression of some diseases.

## 7. Future Directions

As with much obesity research these days, the study raises more questions than it does answers, and it is not entirely clear how these specific mechanisms can be harnessed into a useful clinical treatment. We suggest that whether these mechanisms were identified, researchers could potentially hone in on specific human health outcomes influencing them. From that point, future treatments could conceivably modulate serum cytokines, growth factors to benefit human health.

## Figures and Tables

**Table 1:** Main characteristics of the obese population.

Age (Years)	46 (34–53)	Gender Males/Females *n*	36/44
Obesity classes I/II/III n	8/26/46	CRP	0.56 (0.27–1.3)
MS (APT III) yes/not	51/29	HGF pg/mL	491 (425–565)
MS (ID) yes/not	51/29	IL-12p40 pg/mL	234 (130–317)
HS at US grade1/2/3	22/50/8	IL-6 pg/mL	5.7 (2.3–17-5)
BARD > 2 Males/Females n	9/27	T2DM n	18
SLD at US cm	11 (10.1–12.4)	HOMA	2.75 (1.9–4.7)
BMR kcal/24 h	2220 (1770–2439)	Hypertension n	4
FFM %	56 (49–67)	Cholesterol mg/dL	191 ± 36
FM %	52 ± 7.5	IMT mm	0.9 (0.7–1.1)
RMR/FFM kcal/24 h*kg of body	39 (34–43)	PChe U/L	9605 (8300–10876)
ALT (Males) U/L	36.5 (27–41)	ALT (Females) U/L	23 (17–31)
AST (Males) U/L	24 (21–27)	AST (Females) U/L	20 (16–23)
G-GT mu/L	25 (16–42)	AP mu/L	73 (61–91.5)

Legend to Table 1: MS, metabolic syndrome; HS at US, hepatic steatosis at ultrasonography; SLD, spleen longitudinal diameter; BMR, basal metabolic rate; FFM, free fat mass; FM, fat mass; RMR, resting metabolic rate; HGF, hepatic growth factor; IMT, carotid intima-media thickness; PChe, pseudo cholinesterase; AP, alkaline phosphatase; G-GT, gamma-glutamyl transpeptidase; AST/ALT, transaminases; CRP; C reactive protein; T2DM, type 2 diabetes mellitus.

**Table 2:** Relationships between obesity classes and metabolic syndrome (MS) presence.

Frequency Table	Pearson Chi-Squared = 4.838			Pr = 0.089		
Obesity class	MS					
		Not	Yes	Total		
**I**	n	4	4	8		
	%	13.79	7.84	10.00		
**II**	n	13	13	26		
		**44.83**	**25.49**	46		
**III**	n	12	34	32.50		
	%	**41.3**	**66.67**	57.50		
Total	n	29	51	80		
**Multinomial logistic regression**						
**Number of obs = 80**		**Prob > chi-squared = 0.089**				
d.v.	Obesity class	Coef.	Std. Err.	*z*	*p* >|*z*|	(95% Conf. Interval)
**I**						
i.v.	MS	−1.041	0.782780	−1.33	0.183	−2.576/0.493
**II**						
i.v.	MS	−1.041	0.516	−2.02	0.044	−2.053/0.029
**III**	(base outcome)					

Legend to Table 2: MS, metabolic syndrome. The results of the multinomial logistic regression agree with the column percentages presented by tabulate being the multinomial logistic regression model saturated. Sub-analysing, both models test that the column percentages of obesity classes II are inversely related to the others, regards to the presence of MS, i.e., there were more patients without than with MS, confronted with class III, specified as base outcome; d.v., dependent variable; i.v., independent variable.

**Table 3:** Prediction of indices of indirect calorimetry by spleen longitude diameter (SLD). Prediction of basal metabolic rate (BMR) by IL-6 and IL-12p40 levels.

**Linear Regression, Robust**					
**Number of obs = 80**	***R*-squared = 0.271**				
d.v. BMR	Coef.	Std. Err.	*t*	*p* > |*t*|	(95% Conf. Interval)
i.v. SLD	144.508	27.801	**5.20**	<0.001	89.161/199.855
**Linear Regression, Robust**					
**Number of obs = 80**	***R*-squared = 0.143**				
d.v. FFM	Coef.	Std. Err.	*t*	*p* > |*t*|	(95% Conf. Interval)
i.v. SLD	3.348	1.146	**2.92**	0.005	1.068/5.60
**Linear Regression, Robust**					
**Number of obs = 80**	***R*-squared = 0.006**				
d.v. RMR/FFM	Coef	Std. Err.	*t*	*p* > |*t*|	(95% Conf. Interval)
i.v. SLD	0.3519	0.496	0.71	0.480	−0.636/1.339
**Linear Regression, Robust**					
**Number of obs = 80**	***R*-squared = 0.005**				
d.v. FF	Coef.	Std. Err.	*t*	*p* > |*t*|	(95% Conf. Interval)
i.v. SLD	−0.357	0.503	−0.71	0.479	−1.358/0.643
**Linear Regression, Robust**					
**Number of obs = 78**	***R*-squared = 0.089**				
d.v. BMR	Coef.	Std. Err.	*t*	*p* > |*t*|	(95% Conf. Interval)
i.v. IL-16|	2.117	0323677	**2.89**	0.005	0.658/3.576
**Linear Regression, Robust**					
**Number of obs = 78**	***R*-squared = 0.006**				
d.v. BMR	Coef.	Std. Err.	*t*	*p* > |t|	(95% Conf. Interval)
i.v. IL-2p40	−0.242	0.444	−0.55	0.587	−1.127/0.642

Legend to Table 3: FF, fat mass; FFM, free fat mass; RMR, resting metabolic rate; RMR/FFM, resting metabolic rate/free fat mass ratio; SLD, spleen longitudinal diameter at ultrasonography. The low R-squared, in presence of significance, shows that even noisy, high-variability data can have a significant trend, even though data points fall further from the regression line in graph; d.v., dependent variable; i.v., independent variable. In bold are highlighted the significant ones.

**Table 4:** Prediction of intima-media thickness (IMT) by SLD, fat-free mass (FFM), BMR, FF, fat-free mass (FFM), resting metabolic rate (RMR)/FFM ratio, IL-16 and IL-12p40.

**Linear Regression, Robust**					
**Number of obs = 80,**	***R*-squared = 0.003**				
d.v. IMT	Coef.	Std. Err.	*t*	*p* > |*t*|	(95% Conf. Interval)
i.v. SLD	−0.001	0.002	−0.54	0.594	−0.00/0.003
**Linear Regression, Robust**					
**Number of OBS = 80**	***R*-squared = 0.007**				
d.v. IMT	Coef.	Std. Err.	*t*	*p* > |*t*|	(95% Conf. Interval)
i.v. FFM	0.0002	0.0003	0.72	0.473	−0.0002/0.008
**Linear Regression, Robust**					
**Number of obs = 80**	***R*-squared = 0.0002**				
d.v. IMT	Coef.	Std. Err.	*t*	*p* > |*t*|	(95% Conf. Interval)
i.v. MBR	−1.09 × 10^−6^	8.58 × 10^−6^	−0.13	0.899	−0.00001/0.00002
**Linear Regression, Robust**					
**Number of obs = 80**	***R*-squared = 0.079**				
IMT	Coef.	Std. Err.	*t*	*p* > |*t*|	(95% Conf. Interval)
FF	0.001	0.0005	**2.43**	0.017	0.0002/0.002
**Linear Regression, Robust**					
**Number of obs = 80**	***R*-squared = 0.015**				
IMT	Coef.	Std. Err.	*t*	*p* > |*t*|	(95% Conf. Interval)
RMR/FFM	−0.0006	0.0005	−1.26	0.211	−0.001/0.0003
**Linear Regression, Robust**					
**Number of obs = 78**	***R*-squared = 0.009**				
d.v. IMT	Coef.	Std. Err.	*t*	*p* > |*t*|	(95% Conf. Interval)
i.v. HGF	0.00002	0.00004	0.70	0.488	−0.00005/0.0001
**Linear Regression, Robust**					
**Number of obs = 78**	***R*-squared = 0.0005**				
d.v. IMT	Coef.	Std. Err.	*t*	*p* > |*t*|	(95% Conf. Interval)
i.v. IL-16	0.00001	0.00005	0.26	0.796	−0.00008/0.0001
**Linear Regression, Robust**					
**Number of obs = 78**	***R*-squared = 0.005**				
d.v. IMT	Coef.	Std. Err.	*t*	*p* > |*t*|	(95% Conf. Interval)
i.v. IL-2p40	−0.00002	0.00003	−0.58	0.566	−0.00008/0.00004

Legend to Table 4: FF, fat mass; FFM, free fat mass; RMR, resting metabolic rate; RMR/FFM, resting metabolic rate/free fat mass ratio; SLD, spleen longitudinal diameter at ultrasonography; IMT, carotid intima-media thickness; d.v., dependent variable; i.v., independent variable. In bold is highlighted the significant one.

**Table 5:** Prediction of SLD, IL-16 and IL-12p40 levels by HGF.

**Linear Regression** **, Robust**					
**Number of obs = 78**	***R*-squared = 0.086**				
d.v. SLD	Coef.	Std. Err.	*t*	*p* > |*t*|	(95% Conf. Interval)
i.v. HGF	0.003	0.002	**2.09**	0.04	0.0001/0.006
**Linear Regression** **, Robust**					
**Number of obs = 78**	***R*-squared = 0.095**				
d.v. HGF	Coef.	Std. Err.	*t*	*p* > |*t*|	(95% Conf. Interval)
i.v. IL-16	0.680	0.315	**2.16**	0.034	0.052/1.307
**Linear Regression** **, Robust**					
**Number of obs = 78**	***R*-squared = 0.060**				
d.v. HGF	Coef.	Std. Err.	*t*	*p* > |*t*|	(95% Conf. Interval)
i.v. IL-12p40	0.234	0.137	1.71	0.091	−0.038/0.507

Legend to Table 5: HGF, hepatic growth factor; SLD, spleen longitudinal diameter at ultrasonography; IL-16, interleukin-16; Il-12p40, interleukin-12 subunit p40. The low R squared, in presence of both significant values, shows that even noisy, high-variability data can have a significant trend, even though data points fall further from the regression line in graph; d.v., dependent variable; i.v., independent variable. In bold are highlighted the significant ones.

**Table 6:** Evaluation of the influence of IL-16 in predicting BMR by HGF levels.

Extended Linear Regression					
Number of Obs = 78	Wald Chi-Squared (1) = 4.00		Prob > Chi-Squared = 0.005		
	Coef.	Std. Err.	z	*p* > |*z*|	(95% Conf. Interval)
d.v. BMR					
i.v. HGF	3.114	1.558	**2.00**	0.046	0.061/6.168
i.v. HGF					
e.v IL-16	0.680	0.237	**2.87**	0.004	0.215/1.144
var(e.BMR)	324139.1	164015.7	-----	-----	120232.0/873861.8
var(e.HGF)	16214.72	2596.422	-----	-----	11847.0/22192.7
corr(e.HGF,					
e.BMR)|	−0.6887355	0.1925947	**−3.58**	<0.001	−0.916/−0.127

Legend to Table 6: BMR, basal metabolic rate, HGF, hepatic growth factor; IL-16, interleukin-16. The correlation estimates tells us about the endogeneity in our model of extended linear regression, i.e., IL-16 is an endogenous variable, playing likely a causal role. Although the Prob > chi2 was significant due to the large coefficient, the F-statistic (Wald chi square) against the null, i.e., the excluded endogenous variable are irrelevant in the first-stage regression, should have been larger than 10 to demonstrate that instruments/covariate/endogenous/explanatory variables are weak according to Staiger and Stock’s Rule of thumb; d.v., dependent variable; e.v., endogenous variable; i.v., independent variable. In bold are highlighted the significant ones and the correlation estimates.

**Table 7:** Evaluation of the influence of HGF levels in predicting BMR by SLD.

Regression					
Number of Obs = 78	Wald Chi-Squared (1) = 5.65			Prob > Chi-Squared = 0.017	
	Coef.	Std. Err.	*z*	*p* > |*z*|	(95% Conf. Interval)
d.v. BMR					
i.v. HGF	5.834	2.457	**2.38**	0.017	1.022/10. 653
HGF	0 (omitted)				
i.v. HGF					
e.v. SLD	25.922	9.548	**2.71**	0.007	7.208−44.636
var(e.BMR)	729,014.7	499,010.4	-----	-----	190,585.2/2,788,583.0
var(e.HGF)	16,380.95	2622.96	-----	-----	11,968.58/22,420.0
corr (e.HGF,					
e.BMR)	−0.904	0.071	**−12.79**	<0.001	−0.978/0.627

Legend to Table 7: BMR, basal metabolic rate; SLD, spleen longitudinal diameter at ultrasonography; HGF, hepatic growth factor. The correlation estimates tells us about the endogeneity in our model of extended linear regression, i.e., HGF is an endogenous variable, playing likely a causal role. Although the Prob >chi2 was significant due to the large coefficient, the F-statistic (Wald chi square) against the null, i.e., that the excluded endogenous variable are irrelevant in the first-stage regression, should have been larger than 10 to demonstrate that instruments/covariate/endogenous/explanatory variables are weak according to Staiger and Stock’s Rule of thumb; d.v., dependent variable; e.v., endogenous variable; i.v., independent variable. In bold are highlighted the significant ones and the correlation estimates.

**Table 8:** Prediction of hepatic steatosis (HS) at US by SLD, BMR, IL-16 and IL-12p40.

**Ordered Probit Regression** **, Robust**					
**Number of obs = 80**	**Pseudo *R*-squared = 0.148**				
d.v. HS at US|	Coef.	Std. Err.	*z*	*p* > |*z*|	(95% Conf. Interval)
i.v. SLD	0.428	0.095	**4.48**	<0.001	0.241/0.615
**Ordered Probit Regression** **, Robust**					
**Number of obs = 80**	**Pseudo *R*-squared = 0.112**				
d.v. HS at US	Coef.	Std. Err.	*z*	*p* > |*z*|	(95% Conf. Interval)
i.v. BMR	0.001	0.0003	**3.86**	<0.001	0.0006/0.002
**Ordered Probit Regression** **, Robust**					
**Number of obs = 78**	**Pseudo *R*-squared = 0.005**				
d.v. HS at US	Coef.	Std. Err.	*z*	*p* > |*z*|	(95% Conf. Interval)
i.v. IL-16	0.002	0.002	1.00	0.318	0.002/0.005
**Ordered Probit Regression** **, Robust**					
**Number of obs = 78**	**Pseudo *R*-squared = 0.004**				
d.v. HS at US	Coef.	Std. Err.	*z*	*p* > |*z*|	(95% Conf. Interval)
i.v. IL-12p40	−0.0007	0.0009	−0.81	0.420	−0.002/0.001

Legend to Table 8: HS at US, hepatic steatosis at ultrasonography; BMR, basal metabolic rate; IL-16, interleukin-16; IL-12p40, interleukin-12 subunit p40; SLD, spleen longitudinal diameter at ultrasonography; d.v., dependent variable; i.v., independent variable. In bold are highlighted the significant ones.

**Table 9:** Evaluation of the influence of BMR in predicting HS by SLD.

Extended Linear Regression					
Number of Obs = 80	Wald Chi-Sqaured (1) = 16.21			Prob > Chi-Squared = 0.0001	
	Coef.	Std. Err.	*z*	*p* > |*z*|	(95% Conf. Interval)
d.v. HS at US					
i.v. SLD	0.308	0.076	**4.03**	<0.001	0.158/0.458
i.v SLD	0 (omitted)				
i.v. SLD					
e.v. BMR	0.002	0.0003	**5.45**	<0.001	0.001/0.002
var(e.HS)	0.304	0.067	-----	-----	0.197/0.467

Legend to Table 9: HS at US, hepatic steatosis at ultrasonography; SLD, spleen longitudinal diameter; BMR, basal metabolic rate.

**Table 10:** Evaluation of the influence of Il-6 levels in predicting HS by SLD.

Extended Linear Regression.					
Number of Obs = 80	Wald Chi-Squared (1) = 4.44			Prob > Chi-Squared = 0.035	
	Coef.	Std. Err.	*z*	*p* > |*z*|	(95% Conf. Interval)
d.v. HS at US					
i.v. SLD	7.437	3.528	**2.11**	0.035	0.522/14.353
SLD	0 (omitted)				
i.v. SLD					
e.v. IL-6	0.0003	0.0004	0.71	0.479	−0.0005/−0.001
var(e.HS)	126.483	121.119	-----	------	19.361/826.291
var(e.SLD)	2.397	0.379	-----	-------	1.758/3.268
corr(e.SLD)					
(e.HS)	−0.9989576	0.000985	**−1014.18**	<0.001	−0.999/0.993

Legend to Table 10: The correlation estimates (e.SDL, e.HS) tells us about the endogeneity in our model of extended linear regression, i.e., IL-6 is an endogenous variable, playing likely a causal role. Although the Prob> chi2 was significant due to the large coefficient, the F-statistic (Wald chi square) against the null, i.e., the excluded endogenous variable are irrelevant in the first-stage regression, should have been larger than 10 in order to demonstrate that instruments/covariate/endogenous/explanatory variables are weak according to Staiger and Stock’s Rule of thumb; in our case is <10; d.v., dependent variable; e.v., endogenous variable; i.v., independent variable. In bold are highlighted the significant ones and the correlation estimates. It should be stressed the high value of z in the correlation (e.SLD, eHS).

**Table 11:** Prediction of hepatic steatosis or NAFLD severity using BARD score > 2 by HGF, spleen length, indices of indirect calorimetry.

**Logistic Regression **					
**Number of obs = 78**	**Prob > chi-squared = 0.244**				
d.v. BARD	Odds Ratio	Std. Err.	*z*	*p* > |*z*|	(95% Conf. Interval)
i.v. HGF	0.998	0.002	−1.12	0.261	0.994/1.002
**Logistic Regression **					
**Number of obs = 80**	**Prob > chi-squared = 0.046**				
d.v. BARD	Odds Ratio	Std. Err.	*z*	*p* > |*z*|	(95% Conf. Interval)
i.v. SLD	0.730	0.121	−1.89	0.058	0.527/1.011
**Logistic Regression**					
**Number of obs = 80**	**Prob > chi-squared = 0.023**				
d.v. BARD	Odds Ratio	Std. Err.	*z*	*p* > |*z*|	(95% Conf. Interval)
i.v. BMR	0.999	0.0006	**−2.14**	0.032	0.997/0.999
**Logistic Regression **					
**Number of obs = 80**	**Prob > chi-squared = 0.019**				
d.v. BARD	Odds Ratio	Std. Err.	*z*	*p* > |*z*|	(95% Conf. Interval)
i.v. FFM	0.957	0.019	**−2.16**	0.031	0.920/0.996

Legend to Table 11: HGF, hepatic growth factor; SLD, spleen longitudinal diameter; BMR, basal metabolic rate; d.v., dependent variable; i.v., independent variable. In bold are highlighted the significant ones.
